# Insight into CAZymes of *Alicyclobacillus mali* FL18: Characterization of a New Multifunctional GH9 Enzyme

**DOI:** 10.3390/ijms24010243

**Published:** 2022-12-23

**Authors:** Miriam Carbonaro, Martina Aulitto, Giovanni Gallo, Patrizia Contursi, Danila Limauro, Gabriella Fiorentino

**Affiliations:** 1 Department of Biology, University of Naples Federico II, 80126 Naples, Italy; 2 Biological Systems and Engineering Division, Lawrence Berkeley National Laboratory, Berkeley, CA 94720, USA

**Keywords:** lignocellulose waste, renewable sources, cellulose, *Alicyclobacillus*, thermozymes, CAZymes, multifunctional glycosyl hydrolases

## Abstract

In the bio-based era, cellulolytic and hemicellulolytic enzymes are biocatalysts used in many industrial processes, playing a key role in the conversion of recalcitrant lignocellulosic waste biomasses. In this context, many thermophilic microorganisms are considered as convenient sources of carbohydrate-active enzymes (CAZymes). In this work, a functional genomic annotation of *Alicyclobacillus mali* FL18, a recently discovered thermo-acidophilic microorganism, showed a wide reservoir of putative CAZymes. Among them, a novel enzyme belonging to the family 9 of glycosyl hydrolases (GHs), named AmCel9, was identified; in-depth in silico analyses highlighted that AmCel9 shares general features with other GH9 members. The synthetic gene was expressed in *Escherichia coli* and the recombinant protein was purified and characterized. The monomeric enzyme has an optimal catalytic activity at pH 6.0 and has comparable activity at temperatures ranging from 40 °C to 70 °C. It also has a broad substrate specificity, a typical behavior of multifunctional cellulases; the best activity is displayed on β-1,4 linked glucans. Very interestingly, AmCel9 also hydrolyses filter paper and microcrystalline cellulose. This work gives new insights into the properties of a new thermophilic multifunctional GH9 enzyme, that looks a promising biocatalyst for the deconstruction of lignocellulose.

## 1. Introduction

The realization of an eco-sustainable economy is one of the most urgent current issues of the last few decades, which directs global efforts to reduce CO_2_ emissions and use renewable sources to produce biofuels and bio-products to supplant fossil fuels and petroleum-based products. In this context, lignocellulosic waste is a promising renewable resource owing to its abundance and affordability, and it can be used as a raw material to recover high-value-added products from degradation of its complex structure [1].

The composition of lignocellulose waste is extremely heterogeneous and depends on the waste source (i.e., forestry, agricultural, municipal, industrial) as well as conditions of the plant species such as age, stage of growth and others. The main components of plant biomass are cellulose, hemicellulose and lignin, but mineral residues, proteins, pectins and nitrogenous compounds are also present [2]. Native cellulose is the most abundant homopolymer present in the secondary cell wall of plants; the fundamental repeated unit of the cellulose linear chain is cellobiose, a disaccharide of two D-glucose units 180° rotated to each other and linked through β-1,4-glycosidic bonds. Cellulose chains interact via hydrogen bonds and van der Waals forces and are arranged in microfibrillar superstructures where highly ordered regions (i.e., crystalline) alternate to disordered ones (i.e., amorphous) [3]. As a result of such a structure, cellulose has areas characterized by high fiber strength and low accessibility, making its total decomposition another crucial step in the deconstruction of recalcitrant lignocellulose. The use of biocatalysts for cellulose degradation in biorefineries or other industrial applications has many advantages over chemical hydrolysis, but the costs related to enzyme production constitute one of the main drawbacks; for this reason, the identification of highly active and multifunctional catalysts, that can work skillfully in harsh conditions, is a goal to reduce costs [4,5]. 

The complete degradation of cellulose requires the synergistic action of several enzymes that display activities on different regions of cellulose fibers. They are all named cellulases and are grouped in endoglucanases, exoglucanases and β-glucosidases. Endoglucanases act on internal β-1,4-glycosidic bonds; exoglucanases or cellobiohydrolases hydrolyze the reducing or non-reducing ends of cellulose chains releasing cellobiose which is further cleaved into two glucose units by β-glucosidases. Moreover, lytic polysaccharide monooxygenases catalyze the oxidative cleavage of a glycosidic bond in the presence of hydrogen peroxide or dioxygen [6].

In particular, endoglucanases are glycosyl hydrolases (GHs) with representatives in archaea, bacteria, fungi and eukaryotic organisms and belong to 13 different families of GHs, although the majority are included in families 5, 6, 7, 9, 12 and 45 [7,8].

In the last few decades, thermophilic bacteria have received noticeable attention as reservoirs of thermotolerant, acid and alkali-stable GH enzymes suitable for application in harsh industrial conditions [9,10,11,12,13]. For example, the *Alicyclobacillus* genus embraces strictly aerobic, endospore-forming, thermo-acidophilic gram-positive bacilli, usually thriving in the soil, spoiled fruit-based beverages and hot springs; they possess *ω*-alicyclic fatty acids as the major component of membrane lipids [14]. Recent studies reported that species such as *A. acidocaldarius*, *Alicyclobacillus* sp. A4, *A. vulcanalis*, *A. cellulosilyticus,* etc., are intriguing sources of thermoacidophilic cellulases [15,16]. In this regard, a new strain of *A. mali*, FL18, was recently isolated from a hot mud pool at Pisciarelli, a solfataric field located in the volcanic area of Campi Flegrei near Naples in Italy [17]. 

In this work, we analyzed the lignocellulose degrading potential of *A. mali* FL18 by performing genomic functional annotation to identify the putative catalysts involved in biomass deconstruction. Among the identified coding DNA sequences (CDSs) that were found to be distributed across different CAZymes, we chose to perform more in-depth studies on a GH9 member, AmCel9. The gene was synthetically produced and overexpressed in *Escherichia coli*. The recombinant enzyme was purified and biochemically characterized, revealing activity on a broad range of substrates, pH values and temperatures as well as long-lasting stability at different conditions.

## 2. Results and Discussion

### 2.1. Analysis of CAZymes in A. mali FL18 

**Figure 1:**
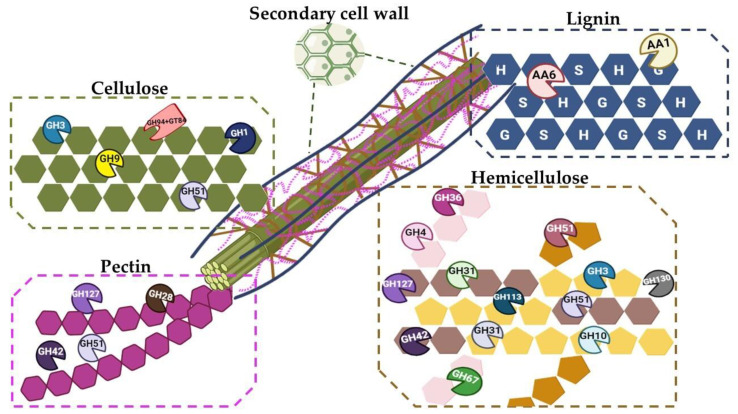
Overview of *A. mali* FL18 GHs on different lignocellulose components. All the GHs revealed by functional annotation are indicated as acting on their preferential lignocellulose substrate.

Extremozymes represent the cornerstone for the development of green, efficient and sustainable technologies [18,19,20,21,22,23]. These biocatalysts, naturally produced by extremophiles and able to work under harsh conditions, are ideal for various industrial processes [2,24,25,26,27,28]. One of the notable applications of these enzymes is their use in the deconstruction of recalcitrant lignocellulosic biomasses [29,30]. Due to the intrinsic features of these enzymes, their industrial market has constantly increased over the years [31]; in the present work, we gain an insight into the CAZymes repertoire of the recently isolated *A. mali* FL18. The prediction obtained through dbCAN 2 metaserver unveiled the presence of 73 CDSs belonging to CAZymes (Table S1) and they correspond to about 2.5% of protein coding genes that are 2832 in the NCBI annotation [32]; this percentage is within the range of the CAZymes encoding genes estimated in the majority of microbial genomes, considering that the CAZomes of microorganisms typically correspond to 1–5% of the predicted CDS [33]. The analysis also showed that the most abundant family predicted in this genome is GH with 32 enzymes, followed by 28 glycosyltransferases (GTs), 11 carbohydrate esterases (CEs) and 2 auxiliary activities (AAs). All the putative genes encoding CAZymes are reported in Table 1 and sketched in Figure 1.

#### Mining of Enzymes for Cellulose Degradation 

The in-depth functional analysis of *A. mali* FL18 genome was performed to identify new cellulose-degrading enzymes for biotechnological purposes (Table 1 and Figure 1). Four members of GHs (GH1, GH9, GH51, GH94) were found; among these, GH1 and GH94 are expected to hydrolyze short oligosaccharides, while GH9 and GH51 are putative endoglucanases. In detail, GH1 (MBF8378818.1) encodes a β-glucosidase [34] and GH94 a putative β-D-glycoside phosphorylases (MBF8378553.1), potentially acting on β-linked substrates such as cellobiose, cellodextrin, laminaribiose, N,N′-diacetylchitobiose and cellobionic acid. This latter enzyme could provide an alternative to cellobiose degradation through the intracellular phosphorolysis [35]. *A. mali* FL-18 genome also encodes two putative arabinofuranosidases (MBF8377873.1 and MBF8377278.1) belonging to the family GH51. In particular, the gene MBF8377873.1, recognized by the dbCAN tool as either putative E.C. 3.2.1.4 (cellulase) or 3.2.1.8 (xylanase), could have a dual role in both cellulose and xylan deconstruction. Indeed, the close relative *A. acidocaldarius* possesses two endoglucanases, CelA (GH9) and CelB (GH51) [36,37], the latter found to be active on both CMC and xylan. Moreover, a recent study has shown that a rumen bacterial strain, *Fibrobacter succinogenes* S85, actively expresses a GH51 endoglucanase 2 (Eg2, CelF, and EgF) and a GH9 endoglucanase 1 (Cel2 and End1) in the presence of cellulosic material [38]. 

Interestingly, one putative endoglucanase member of the GH9 family, MBF8377998.1, was also found. The GH9 family comprises some of the most processive and multicatalytic β-1,4 endoglucanases, mixed-linkage endoglucanases, cellobiohydrolases and endo-xyloglucanases, primarily from bacteria [39]; they all function by hydrolyzing the glycosidic bond with an inverting mechanism (http://www.cazy.org/GH9.html, accessed on 10 November 2022). GH9 members generally possess a catalytic GH domain (with an α/α 6 barrel-fold containing the active site cleft) and at least one accessory module, such as carbohydrate-binding modules (CBM, that allow binding to insoluble polysaccharides and seem to be related to enzymatic processivity) or Ig-like domains [40,41]. Despite the conservation of aminoacidic sequence, it has not been possible to define a classification of modular structures for this family [39,40]. In fact, a recent analysis of GH9 sequences derived from bacterial metagenomes showed the presence of 19 different modules. Interestingly, only 9% of GH9 members shared five modules, revealing that this family has a more heterogeneous architecture of domains. It has been proposed that such a heterogeneous structure might reflect their ability to work as multifunctional enzymes that can either accommodate various polysaccharides in their active site cleft or exhibit improved activity on the native substrate through increased processivity [4]. The discovery of multifunctional cellulases with different hydrolytic capabilities has an important outcome in the biotechnological application, for the improvement of the enzyme-assisted steps in the lignocellulose biorefinery process. For these reasons, the GH9 protein MBF8377998.1 of 537 aa identified was chosen for in-depth studies; it was named AmCel9.

### 2.2. Sequence Analysis of AmCel9

**Figure 2:**
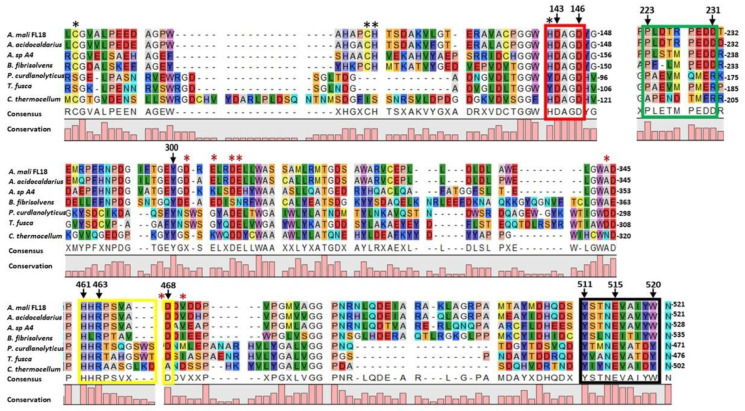
Multiple sequence alignment of AmCel9 aminoacidic sequence (from aa 103 to 521) with other GH9 family members realized with CLC Main Workbench 22.0.2. Red and black boxes highlight conserved catalytic residues; the yellow and green boxes indicate the regions involved in the interaction with Ig-like domain and substrate; the black and red asterisks mark the aa that interact with zinc and calcium ions, respectively. The highly conserved amino acid residues are indicated by arrows.

Using the AmCel9 amino acid sequence as query, BLASTp was used to find sequences with a percentage of identity ranging from 20% to 90%. A multiple alignment was then obtained with prokaryotic enzymes already characterized, i.e., those from *A. acidocaldarius* (89.3%; CelA; Accession No. WP_012811722.1), *Alicyclobacillus* sp. A4 (48.4%; Alg9; Accession No. ADH59533.1), *Butyrivibrio fibrisolvens* (34.3%; CED1; Accession No. WP_026661505.1), *Paenibacillus curdlanolyticus* (22.5%; PcMulGH9; Accession No. WP_127568011.1), *Thermomonospora fusca* (24.4%; E4; Accession No. WP_011292599.1) and *Clostridium thermocellum* (23.7%; Cel9T; Accession No ABN54011.1) (Figure 2). 

In the multiple alignment, the red box shows a consensus motif Y/HDAGD typical of GH9 members encompassing the two catalytic aspartic residues (143–146 in AmCel9). Additionally, catalytic glutamate E515 in AmCel9, with a key role as proton donor, is included within a highly conserved motif Y–NEVA-Y/DW/Y (highlighted in the black box, region from aa 511 to 520). The conserved residues Y511, W520 and Y300, as in Cel9A, could be essential to guarantee the right orientation of glutamate to correctly bind the substrate [37]. In the AmCel9 sequence, the residues that in Cel9A are also involved in zinc and calcium binding are conserved; in fact, these ions form coordination bonds with the residues in the catalytic domain labeled with red and black asterisks (Figure 2), contributing to improved thermostability or enzyme substrate affinity [42]. AmCel9 contains a N-terminal Ig-like domain of 85 aa followed by a C-terminus catalytic domain and lacks CBM modules, that are present at the C-terminus of the GH9s of *P. curdlanolyticus* and *T. fusca* [37,43].

To date, the function of the Ig-like domain is not completely clear, but previous studies highlighted its importance in promoting the right folding of the catalytic center or the substrate binding site [44], as for example in CelA of *A. acidocaldarius* where it contributes to enzyme stability during cellulose binding at high temperatures [42]. A recent phylogenetic study has been performed to try to better clarify the role of this module; since the Ig-like domain is found only in some members of bacterial GH9s, it has been proposed that a common ancestor probably existed, which was lost early during evolution. On the other hand, the presence or absence of other accessory domains (CBM2, CBM3, CBM4/9, dockerin) seems to be independent on the phylogenetic clustering and only the upstream Ig-like module has probably co-evolved with the catalytic domain in the enzymes that maintained it [40]. The alignment shown in Figure 2 supports this hypothesis; in fact, the first four proteins bringing the Ig-like domain at the N-terminus also display the highest similarity in the aminoacidic sequence of the catalytic regions. Moreover, in the characterized Cel9A of *A. acidocaldarius* a dynamic interaction has been evidenced between residues of the Ig-like module and the regions of the catalytic domain corresponding to the “green box” PL—PEDD, and “yellow box”, HHRPSVX—and amino acids Y437 and N442 (Figure 2) [42]. It is noteworthy that all the proteins in the alignment contain the yellow box, whereas only the proteins with a N-terminus Ig-like domain (the first four) share the “green box”; for this reason, we also hypothesize that AmCel9 the “green box” of the catalytic domain could interact with the Ig-like domain whereas the “yellow box”, could be somewhat implied in substrate binding [37]. 

**Figure 3:**
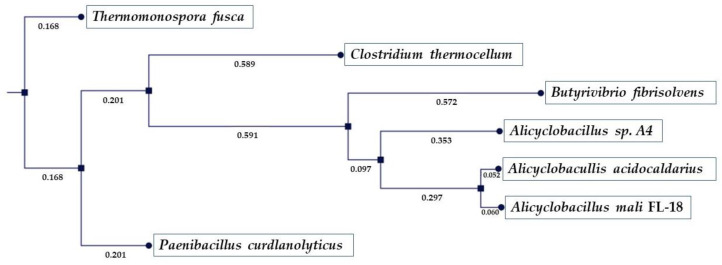
Phylogenetic tree by CLC Main Workbench 22.0.2. The numbers on the nodes correspond to the percentage of bootstrap values for 200 replicates.

Multiple alignment was also used to construct an evolutionary tree to depict the phylogenetic relationship among the proteins investigated (Figure 3). 

The topology of the tree classifies the sequences according to their differences, reflecting their evolutionary distances; in fact, considering the sequence of *A. mali* FL18 as input, the one of *T. fusca* is the farthest from an evolutionary point of view. As expected, the closest is that of *A. acidocaldarius* which shares the highest number of identical aa.

**Figure 4:**
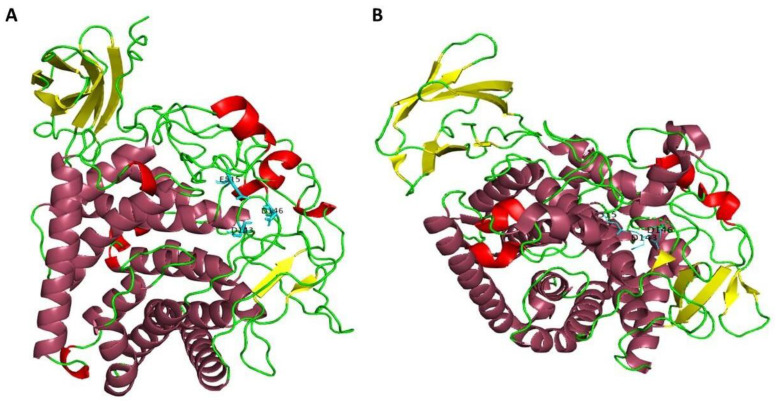
Structural comparison between the predicted 3D structure of AmCel9 (**A**) and the crystal structure of Cel9A of *A. acidocaldarius* (**B**) [45]. For both, the N-terminus Ig-like domain (yellow) is made up of seven β-sheets; the catalytic triad (light blue) is placed in the cleft of the catalytic (α/α)_6_ barrel domain (purple).

To investigate the structural features of AmCel9, a 3D model of the enzyme was obtained using ColabFold and the obtained structure (Figure 4A) was compared to the crystal structure of Cel9A of *A. acidocaldarius* (Figure 4B) [45]. 

The two structures resulted in being very similar to each other, both characterized by the Ig-like domain at the N-terminus and the catalytic domain with an (α/α)_6_ barrel motif at C-terminus. In AmCel9, the Ig-like domain and catalytic domain are linked by a longer loop region near the substrate binding cleft in the three-dimensional structure, probably connected with the endo-activity efficiency [40]. Indeed, Figure 4 shows that the *A. mali* structure lacks two β-sheets connected to the catalytic region that are replaced by a longer loop region; accordingly, E515 is also placed in this region and not in a α-helix [40].

### 2.3. Recombinant AmCel9: Expression, Purification, and Molecular Weight Analyses

The recombinant N-terminal His-tagged enzyme AmCel9 was expressed in *E. coli* BL21-CodonPlus(DE3) cells transformed with the plasmid pET28b(+)/*AmCel9*. The overexpression was induced in the recombinant cells with 1 mM of isopropyl-1-thio-β-d-galactopyranoside (IPTG), at 37 °C for 16 h, conditions of concentration, temperature and time that resulted in being optimal for the high-level expression of the recombinant protein. Enzyme purification to homogeneity was achieved by a two-step purification procedure (i.e., thermal precipitation at 60 °C and affinity chromatography through a His-trap column), and purified protein was visualized on a SDS-PAGE gel as a band migrating at the expected molecular weight of ~59 kDa (Figure S1 (Supplementary Materials)). The final protein yield was 16 mg/L. 

To investigate the quaternary structure of AmCel9, gel-filtration chromatography coupled with a triple-angle light scattering QELS was performed. The results showed that the enzyme in solution has a molecular weight of 58.81 kDa ± 1% (Rh = 7.2 nm ± 3%) indicating that AmCel9 is a monomer resembling the enzyme CelA of the close relative *A. acidocaldarius* (Figure S1) [45]. 

### 2.4. pH and Temperature Profile and Stability Properties of AmCel9

**Figure 5:**
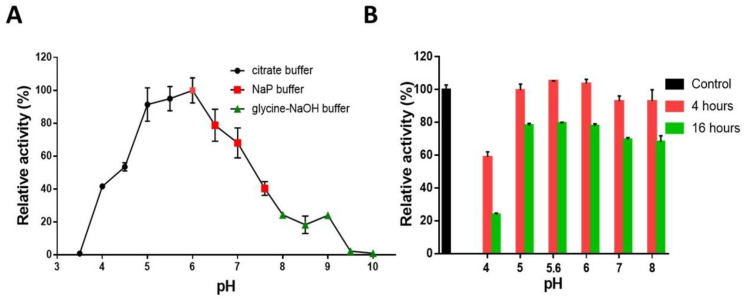
Effect of pH on activity (**A**) and stability (**B**) of AmCel9. (**A**) Black dots indicate the percentage of activity in citrate buffer (pH from 3.5 to 5.5); red squares in Na-P buffer (pH from 6 to 7.5) and green triangles in glycine-NaOH buffer (pH from 8 to 10). (**B**) Relative activities at the optimal pH 6 without incubation (control, black), and after 4 and 16 h of incubation at different pH values (red and green, respectively).

To assess the enzymatic activity at different pH values and temperatures, AmCel9 activity was assayed using CMC as the substrate. Although the pH optimum for *A. mali* FL18 growth is pH 4, the purified AmCel9 showed its pH optimum at pH 6.0 and displayed more than 50% activity within the pH range from 5.0 to 7.0 (Figure 5A).

The tolerance within this range of pH is a feature already observed in other characterized processive GH9s and the instability at low pH values could be related to the localization of AmCel9 in the pH-neutral cytoplasm, as also suggested by the lack of a signal sequence for secretion [46,47]. AmCel9 also displayed a significant long-lasting stability at different pH values; indeed, at pH values from 5.0 to 8.0 (Figure 5B) no loss of activity was observed after 4 h of incubation at 4 °C, whereas a residual activity up to 60% was preserved at pH 4.0. Interestingly, after 16 h of incubation at 4 °C, even though the activity drastically decreased at pH 4.0, it was still about 70% at pH values from 5.0 to 8.0. This stability is a peculiar feature since other GH9 members, i.e., Agl9A of *Alicyclobacillus* sp. A4 or CenC of *C. thermocellum*, lost their activity after a few minutes of preincubation at different pH values [48,49]. 

**Figure 6:**
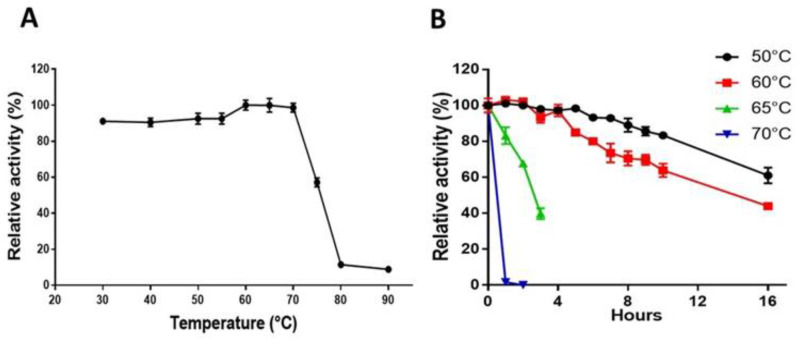
AmCel9 catalytic activity at different temperatures (**A**) and thermo-resistance in the range of 50 °C to 70 °C (**B**).

It is of note that the temperature activity profile (Figure 6A) revealed that at pH 6.0 AmCel9 has almost 100% activity at temperatures ranging from 40 °C to 70 °C, and retains more than 50% of activity at 75 °C, a feature also found in Agl9A of *Alicyclobacillus* sp. A4 and in two GH9s of *C. thermocellum* (Cel9T and Cel9D) [46].

Regarding the thermo-resistance, the enzyme retained more than 60% and 40% of activity after being incubated at 50 °C and 60 °C for 16 h, respectively, therefore showing good stability (Figure 6B). The comparison of this behavior with the data from the literature is noticeable; for example, the endoglucanases CelA of *A. acidocaldarius* and CenC of *B. licheniformis* despite having their temperature optimum at 70 °C, lost more than 40% of their activity after 2 h of incubation at 60 °C. Moreover, *Bl*Cel9 of *B. licheniformis* also showed long-lasting thermostability at 50 °C, retaining more than 80% of its activity after 24 h; however, at 60 °C, its enzymatic activity dropped significantly to about 40% after only 3 h, whereas AmCel9 was still over 60% active after 10 h [50]. These data suggest that AmCel9 may be considered as an appealing potential biocatalyst in several bioprocesses such as pretreatment of lignocellulosic wastes, requiring high temperatures that are known to favor degradation and substrate solubility; other industrial processes in which pH values and temperature vary, as for example in brewing industry where the use of β-glucanases and β-xylanases (at pH 5.0–6.0 and at 45–65 °C) resulted in an efficient decrease in the viscosity and filtration time [30,51]. 

### 2.5. Substrate Specificity and Catalytic Properties 

GH9 enzymes generally exhibit heterogeneous hydrolytic capabilities on cellulose; most of them are mainly endoglucanases such as the endo β-1,4-glucanase Cel9A of *A. acidocaldarius* or the endo-β-1,3(4)-glucanase of *Alicyclobacillus* sp. A4 [37,48], but others show exoglucanase activity such as the cellobiohydrolases CbhA and CelK isolated from *C. thermocellum* [52,53]. This family of GHs also includes enzymes that possess multiple catalytic activities, for example, the E4 of *T. fusca* with endo and exo-activities; or bifunctional Cel9X of *C. cellulolyticum* with cellulase/xylanase activities; or the cellulase/mannanase/xylanase activities of *P. curdlanolyticus* B-6 that hydrolyze different polysaccharides [43,54,55].

To assess whether AmCel9 also had a heterogeneous activity towards various substrates, different soluble polysaccharides were tested; the high viscosity of the soluble substrate precluded their preparation at concentrations higher than those utilized (see Section 3). Under these conditions, the best activity displayed by the enzyme was on β-1,4-linked glucans i.e., CMC (512.7 ± 2.0 U/mg) and lichenan (1115 ± 0.01 U/mg); therefore, these substrates were used to evaluate the kinetic parameters of AmCel9. Complete substrate saturation could not be obtained because of the high viscosity of the substrates; hence, the *K*_M_ and *V*max values (Table 2) were estimated fitting the data to Michaelis–Menten curve by GraphPad Prism and calculating them from the extrapolated graph (Figure S2).

The AmCel9 kinetic parameters indicate a very efficient enzyme on both substrates (Table 2); in fact, results show a high affinity of AmCel9 for CMC and lichenan with a Michaelis constant (*K*_M_) comparable with those determined for other GH9s [8]. The turnover number (k_cat_) calculated for lichenan was about two-fold higher than that on CMC. Therefore, the k_cat_/*K*_M_ of AmCel9 on this soluble substrate is about three times lower than that on lichenan; the lower enzyme activity could be due to the presence of methyl groups on CMC side chains. Overall, since most other GH9 members have not been characterized to date, the determination of the kinetic parameters of AmCel9 gives new insight into the biochemical features of GH9 family [8].

Unfortunately, an all-embracing comparison of activity values within characterized GH9 members is not feasible because of their heterogeneity as each protein has different specific activity and substrate specificity on several polysaccharides [44]. However, we observed that specific activity of AmCel9 is much higher than that reported for *A. acidocaldarius* (73 U/mg and 109 U/mg on CMC and lichenan, respectively) [37]. In addition, the endoglucanase of *C. thermocellum* (30 U/mg on CMC) [49], GH9b and GH9d enzymes (3.94 U/mg and 1.46 U/mg on CMC, respectively) [40], Alg9 of *Alicyclobacillus* sp. A4 (33.2 U/mg on lichenan) [48] and PcMulGH9 (0.652 U/mg on CMC) have much lower specific activities than AmCel9 [43]. Interestingly, AmCel9 exhibited high activity towards KGM (1330 ±0.012 U/mg), an unusual characteristic for the GH9 members which hydrolyze different types of mannans but with lower specific activity [40,43], and it also showed low hydrolytic activity towards larch arabinogalactan (2.3 ± 0.2 U/mg) and wheat arabinoxylan (1.7 ± 0.6 U/mg). On the other hand, under the same experimental conditions, no activity was detectable on locust-bean gum mannan, guar galactomannan, carob galactomannan, birchwood xylan, beechwood xylan, laminarin, salicin or cellobiose. Overall, the results suggest that AmCel9 is mainly able to hydrolyze β-1,4-glycosidic bonds, and it does not possess significant β-glucosidase activity. Moreover, since AmCel9 possesses β-1,4-endoglucanase activity with three polysaccharide types it can be considered to be a multifunctional cellulase according to a recent definition proposed by Glasgow et al. [4].

Family GH9 also embraces catalysts that are able to degrade insoluble cellulose; to date, the presence of CBM modules is known to be correlated to the enzyme processivity on microcrystalline cellulose [4,40]; for example, PcMulGH9 of *P. curdlanolyticus* and *Bl*Cel9 of *B. licheniformis*, both harboring CBM modules, have specific activities towards Avicel of 28.57 U/g and 40 U/g, respectively [43,50]. On the other hand, enzymes that share the same modular structure of AmCel9 (Ig-like and GH domains) have much lower or a lack of activity on microcrystalline cellulose as in the case of CelA of *A. acidocaldarius*, Agl9A of *Alicyclobacillus* sp. A4 or BP_Cel9 [37,44,48]. Very interestingly, the hydrolytic activity of AmCel9 evaluated on circle paper units of 0.5 mm × 0.5 mm (Whatman^®^ filter paper) and microcrystalline cellulose (Avicel PH101), showed a good enzyme capability to release glucose with values of specific activity of 68.7 ± 0.02 and 62.98 ± 0.05 U/g, respectively, comparable to those reported for PcMulGH9 or *Bl*Cel9 [43,50]. It can be hypothesized that the high activity observed towards heterogeneous solid Avicel could be due to endoglucanase activity on the amorphous cellulose areas of the substrate [56].

The unexpected activity towards insoluble cellulose along with the broad substrate specificity and high catalytic efficiency make AmCel9 a promising multifunctional catalyst, that might more efficiently break down lignocellulosic material without requiring non-eco-friendly pre-treatments.

## 3. Materials and Methods

### 3.1. Functional Annotation of A. mali FL18 CAZymes

The genome of *A. mali* FL18 was previously annotated by the Rapid Annotation Subsystem Technology (RAST) and deposited in NCBI (JADPKZ000000000) and IMG-IM (https://img.jgi.doe.gov/cgi-bin/mer/main.cgi?section=TaxonDetail&page=taxonDetail&taxon_oid=2931470914 accessed on 10 November 2022). CAZyme searches were performed using dbCAN metaserver (https://bcb.unl.edu/dbCAN2/ accessed on 10 November 2022) with integrated: (i) HMMER database; (ii) DIAMOND for fast blast hits in the CAZy database and (iii) HMMER for dbCAN-sub [32]. 

### 3.2. Reagents and Substrates

Carboxymethyl cellulose sodium salt (CMC), Avicel (PH-101), glucose, cellobiose, salicin, locust bean gum mannan and laminarin were purchased from Sigma-Aldrich (St. Louis, MO, USA). Konjac glucomannan (KGM), birchwood xylan, beechwood xylan, lichenan, guar galactomannan, carob galactomannan, larch arabinogalactan and wheat arabinoxylan were purchased from Megazyme (Bray, Co. Wicklow, Ireland). Whatman^®^ filter paper (75 mm × 100 mm) was cut into circle paper units of 0.5 mm × 0.5 mm. Kanamycin and chloramphenicol were purchased from PanReac AppliChem (Ottoweg, Darmstadt, Germany).

### 3.3. AmCel9 In Silico Analyses 

The aminoacidic sequence of the putative GH9 of *A. mali* FL18, with the Accession No. MBF8377998.1, was compared to sequences of GH9s already characterized available in the GeneBank database by utilizing BLASTp server (https://blast.ncbi.nlm.nih.gov/Blast.cgi accessed on 14 September 2022).

To identify homologous proteins and conserved domains in AmCel9, a multiple alignment and a phylogenetic tree were performed with CLC Main Workbench 22.0.2 software (QIAGEN, Hilden, Germany) by using the amino acid sequences of: Cel9A from *A. acidocaldarius*; Alg9 from *Alicyclobacillus* sp. A4; CED1 from *B. fibrisolvens*; PcMulGH9 from *P. curdlanolyticus*; E4 from *T. fusca* and of Cel9T from *C. thermocellum* [37,43,46,48,54]. A 3D model of AmCel9 was generated using the Colab server (https://colab.research.google.com/github/sokrypton/ColabFold/blob/main/AlphaFold2.ipynb accessed on 14 September 2022); this server predicts protein structures from their sequences using a simplified version of AlphaFold v2.0 [57].

### 3.4. Expression and Purification of Recombinant AmCel9 

The plasmid pET28b(+)/*AmCel9*, which contains the gene encoding AmCel9 with a six-His tag at its N-terminus, was purchased from GenScript Biotech (Piscataway, NJ, USA) and used to transform *E. coli* BL21-CodonPlus(DE3) cells (Stratagene, San Diego, CA, USA). These cells were cultured in LB medium supplemented with kanamycin (50 μg/mL) and chloramphenicol (33 μg/mL). Protein expression was induced for 16 h by adding 1 mM of IPTG (PanReac AppliChem, Ottoweg, Darmstadt, Germany) when the cultures reached 0.7 OD_600_/mL. Cells were harvested by centrifugation at 6750× *g* and resuspended in 20 mL of buffer A (50 mM Tris-HCl pH 8, 300 mM NaCl, 20 mM imidazole, DTT 1 mM) supplemented with lysozyme 0.2 mg/mL (PanReac AppliChem, Ottoweg, Darmstadt, Germany) and with protease inhibitor cocktail 1X (Roche, Basilea, Switzerland). The resuspended cells were lysed with one cycle of freeze-thaw with the following procedure: (i) incubation for 90 min on an orbital shaker at 37 °C; (ii) freezing with ethanol and dry ice for 15 min and (iii) re-incubation for 15 min at 37 °C. After a centrifugation step at 35,000× *g* for 45 min at 4 °C, the purification of AmCel9 was set up through a two-step procedure. A first thermal precipitation at 60 °C for 10 min was followed by centrifugation at 35,000× *g* for 45 min at 4 °C then followed by affinity chromatography on a His-Trap™ column (1 mL, GE Healthcare, Chicago, IL, USA) connected to an AKTA Explorer system. The elution was performed with buffer A supplemented with a linear gradient of imidazole (0–500 mM), and all the peak fractions were pooled and then dialyzed against a storage buffer (50 mM of Tris-HCl pH 8, 300 mM of NaCl and 1 mM of DTT) using an Ultracel^®^ 10 kDa ultrafiltration disc (Sigma-Aldrich, St. Louis, MO, USA) connected to an Amicon^®^ stirred cell. Bradford assay was used to estimate protein concentration using bovine serum albumin as standard. The purity degree of AmCel9 were appraised by SDS-PAGE analyses (12%) after staining the gel with Coomassie brilliant blue R-250 (Biorad, Hercules, CA, USA).

### 3.5. Determination of AmCel9 Native Molecular Weight

The purified AmCel9 was further analyzed by using a size-exclusion chromatography system connected to Mini DAWN Treos light-scattering system (Wyatt Technology, Santa Barbara, CA, USA) equipped with a QELS module (quasi-elastic light scattering module) and with mass value and hydrodynamic radius (Rh) measurements. One milliliter of protein (0.6 mg/mL) was loaded on a Superdex S200 column (10/300; GE Healthcare, Chicago, IL, USA) equilibrated in buffer 50 mM Tris-HCl pH 8, 200 mM NaCl, 1mM DTT, with a flowrate of 0.5 mL/min. Data were analyzed using Astra 5.3.4.14 software (Wyatt Technology, Santa Barbara, CA, USA) [58].

### 3.6. Enzyme Assay, Temperature and pH Profile

AmCel9 standard assay was set up at 50 °C and using CMC as substrate; the assay mixture (total volume of 40 µL) contained 1.6% (*w*/*v*) CMC in assay buffer (50 mM Na-P pH 6, 200 mM NaCl and 3 mM DTT). A total of 1 µg of enzyme was added to the substrate mix, pre-incubated for 10 min at 50 °C and the reaction stopped after 1 min at 50 °C in ice for 5 min. Then, 160 µL of 3,5-dinitrosalicylic acid (DNS) was added to the reaction mix, which was transferred in a 96-well microplate and placed in a thermomixer (Eppendorf, Hamburg, Germany) at 100 °C for 20 min [59,60]. After a cooling step at 4 °C of 20 min, the concentration of reducing sugars in solution was determined by measuring A_540nm_ in a 96-well microplate reader (Synergy H4, Biotek, Agilent, Santa Clara, CA, USA) and by interpolating absorbance data with a standard curve that was prepared according to Kim et al. [59]. One unit of enzyme activity (U) was defined as the amount of enzyme required to release 1 μmol of glucose-reducing equivalent in one minute, under the described assay conditions. 

The optimal pH was determined by measuring enzyme activity in different buffers from pH 3.0 to 10.0 (each 50 mM), with increments of 0.5 pH units at 50 °C for one minute, using the DNS assay described above. Citrate buffer (pH 3.0–5.5), Na-P buffer (pH 6.0–7.5) and glycine-NaOH buffer (pH 8.0–10) were used. Temperature optimum was measured by assaying enzyme activity at pH 6.0 from 40 °C to 90 °C with an increase of 10 °C; each experiment was performed in triplicate and results were reported as relative activity (%). 

To test the pH stability of recombinant AmCel9, it was preincubated in various buffers from pH 4.0 to 8.0 at 4 °C for 4 and 16 h; residual activity of the enzyme was measured under standard assay conditions. Thermo-resistance was determined by measuring residual activity up to 16 h (at 1-h intervals), pre-incubating the purified enzyme without the substrate at temperatures of 50 °C, 55 °C, 60 °C, 65 °C and 70 °C. All the experiments were performed in triplicate and the 100% was the enzyme activity in the absence of pre-incubation.

### 3.7. Substrate Specificity and Kinetic Studies

The substrate specificity of AmCel9 was tested by using the standard assay (1 µg of enzyme, at 50 °C and pH 6 for 1 min) on several polymers: cellobiose (1.5%), lichenan (1.4%), laminarin (1.5%), locust bean gum mannan (0.5%), KGM (0.7%), birchwood xylan (0.9%), beechwood xylan (0.9%), guar galactomannan (0.7%), carob galactomannan (0.7%), larch arabinogalactan (0.7%) and wheat arabinoxylan, prepared in assay buffer (50 mM Na-P pH 6, 200 mM NaCl and 3 mM DTT) or in water, according to the specific data-sheet of each substrate. The released reducing monosaccharides were quantified using the DNS method described above, except for cellobiose, for which the amount of free glucose was estimated by using the D-Glucose Assay Kit (GOPOD Format, Megazyme, Bray, Co. Wicklow, Ireland) according to the manufacturer’s protocol. The assay on CMC (1.6%) was used as the control; one unit (U) was defined as the amount of enzyme necessary to release 1 μmol of reducing sugars per minute at 50 °C and pH 6; each experiment was performed in triplicate.

The kinetic parameters were determined using CMC and lichenan as substrates: 0.4 μg of AmCel9 was tested with CMC (0.4%, 0.6%, 0.8%, 1%, 1.2%, 1.4%, 1.6%) and 0.2 μg with lichenan (0.2%, 0.4%, 0.6%, 0.8%, 1%, 1.2%, 1.4%). The Michaelis–Menten constant K_M_ was calculated by non-linear regression analysis using GraphPad 7.0 (Prism software) and each experiment was performed in triplicate.

The hydrolytic activity of the enzyme was also investigated on insoluble celluloses; the assays were performed in a total volume of 500 μL, by incubating 2.5 mg of Avicel (PH-101) or 1 circle paper unit of 0.5 mm × 0.5 mm (Whatman^®^ filter paper) with 35 μg of purified enzyme in the assay buffer (50 mM Na-P pH 6, 200 mM NaCl and 3 mM DTT) at 55 °C for 1 h on an orbital shaker at 200 rpm. To measure the amount of glucose released, 500 μL of DNS solution were added to each sample and the mixture boiled for 10 min. After a cooling step at 4 °C for 30 min, the A_540nm_ was measured and data were interpolated with a glucose standard curve to measure the concentration of the reducing sugar in solution [49,61]. For each reaction, a non-enzyme control was set up and each experiment was performed in quadruplicate.

## 4. Conclusions

In this study, the potential of the thermoacidophilic bacterium *A. mali* FL18 as a reservoir of CAZymes was investigated. Genome-wide analysis and functional annotation were performed to identify the key contributors in the lignocellulose decomposition and to provide insight into the genes correlated with lignocellulolytic activity. In particular, the analysis of CAZome revealed the presence of 32 putative GHs, indicating that *A. mali* FL18 possesses a palette of interesting activities for the biodegradation of the holocellulosic component of the cell wall. In silico analyses suggest that at least four of these GHs might be involved in cellulose degradation; among them, a novel thermophilic GH9, AmCel9, was biochemically characterized. AmCel9 exhibits a peculiar activity in a broad range of temperatures, as well as thermo-resistance and stability in a wide range of pH values. Moreover, AmCel9 hydrolyses β-glycosidic linkages of synthetic and natural celluloses with relevant specific activities, and, to the best of our knowledge, is the first GH9 member without a CBM module that is also able to hydrolyze insoluble celluloses. Overall, the data presented in this work prove that AmCel9 is a catalyst deserving further exploitation; in fact, it can be employed either as multifunctional enzyme to ameliorate the degradation of lignocellulosic biomass for the conversion of recalcitrant residues into value-added products or to specifically hydrolyze crystalline cellulose.

## Figures and Tables

**Table 1:** *A. mali* FL18 predicted proteins containing CAZymes modules acting on lignocellulose components identified through dbCAN. Protein accession numbers, potential EC number(s), NCBI annotation and the CAZy family are shown.

Accession Number	E.C. Number	NCBI Annotation	CAZy Family
**Cellulose**			
MBF8378818.1	3.2.1.21|3.2.1.-|3.2.1.38|3.2.1.23|3.2.1.74	β-glucosidase	GH1
MBF8377998.1	3.2.1.4|3.2.1.6|3.2.1.151	glycoside hydrolase family 9 protein	GH9
MBF8377873.1	3.2.1.4|3.2.1.8	α-l-arabinofuranosidase	GH51
MBF8378553.1	2.4.1.333|2.4.1.-|-	carbohydrate-binding protein	GH94 + GT84
**Hemicellulose**			
MBF8378422.1	3.2.1.37|3.2.1.55|3.2.1.21	glycoside hydrolase family 3 C-terminal domain-containing protein	GH3
MBF8376310.1	3.2.1.22	α-glucosidase/α-galactosidase	GH4
MBF8379065.1	3.2.1.8	endo-1,4-β-xylanase	GH10
MBF8377308.1	3.2.1.20|3.2.1.10	α-glucosidase	GH31
MBF8377285.1	3.2.1.177	α-xylosidase	GH31
MBF8378558.1	3.2.1.22	α-galactosidase	GH36
MBF8377281.1	3.2.1.23	β-galactosidase	GH42
MBF8377873.1	3.2.1.4|3.2.1.8	α-l-arabinofuranosidase	GH51
MBF8377278.1	3.2.1.55	α-*N*-arabinofuranosidase	GH51
MBF8377854.1	3.2.1.139|3.2.1.131	α-glucuronidase	GH67
MBF8378555.1	3.2.1.78|3.2.1.25	1,4-β-xylanase	GH113
MBF8376699.1	3.2.1.185	glycoside hydrolase family 127 protein	GH127
MBF8377082.1	2.4.1.339	glycoside hydrolase family 130 protein	GH130
MBF8376362.1	-	polysaccharide deacetylase family sporulation protein PdaB	CE4
MBF8376532.1	-	polysaccharide deacetylase family protein	CE4
MBF8377192.1	-	polysaccharide deacetylase family protein	CE4
MBF8377262.1	-	polysaccharide deacetylase family protein	CE4
MBF8377649.1	-	polysaccharide deacetylase family protein	CE4
MBF8378334.1	-	polysaccharide deacetylase family protein	CE4
MBF8377707.1	-	N-acetylglucosamine-6-phosphate deacetylase	CE9
MBF8376962.1	-	PIG-L family deacetylase	CE14
MBF8377409.1	3.5.1.-	PIG-L family deacetylase	CE14
MBF8377724.1	-	bacillithiol biosynthesis deacetylase BshB2	CE14
**Pectin**			
MBF8377980.1	-	glycoside hydrolase family 28 protein	GH28
MBF8377281.1	3.2.1.23	β-galactosidase	GH42
MBF8377278.1	3.2.1.55	α-*N*-arabinofuranosidase	GH51
MBF8376699.1	3.2.1.185	glycoside hydrolase family 127 protein	GH127
**Lignin**			
MBF8377978.1	1.10.3.2	multicopper oxidase domain-containing protein	AA1
MBF8377540.1	-	NAD(P)H:quinone oxidoreductase type IV	AA6

**Table 2:** Kinetic parameters of AmCel9 on two substrates. Activity was measured under optimal conditions (pH 6 and temperature of 50 °C) for 1 min.

Substrate	*K*_M_ (mg mL^−1^)	k_cat_ (s^−1^)	k_cat_/*K*_M_ (mL mg^−1^ s^−1^)
CMC	10.9	847.2	77.7
Lichenan	7.5	1626.5	216.9

## Data Availability

Not applicable.

## Supplementary Materials

The following supporting information can be downloaded at: https://www.mdpi.com/article/10.3390/ijms24010243/s1.
