# Supplementary figures and images for: Insight into CAZymes of Alicyclobacillus mali FL18: Characterization of a New Multifunctional GH9 Enzyme
Source: Int J Mol Sci. 2022 Dec 23;24(1):243. doi: 10.3390/ijms24010243 (PMC9820247; doi:10.3390/ijms24010243)

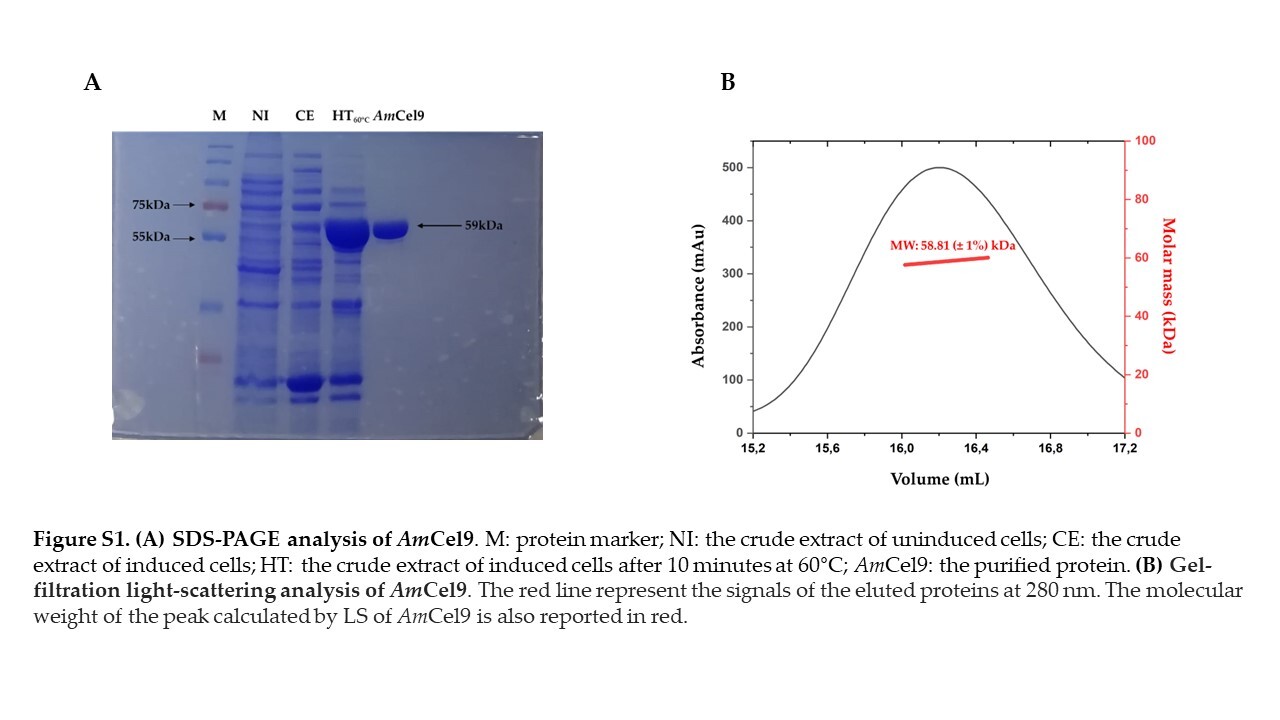

Supplement: Supplementary file 1 [file ijms-24-00243-s001.zip › Supplementary Figure S1.jpg]

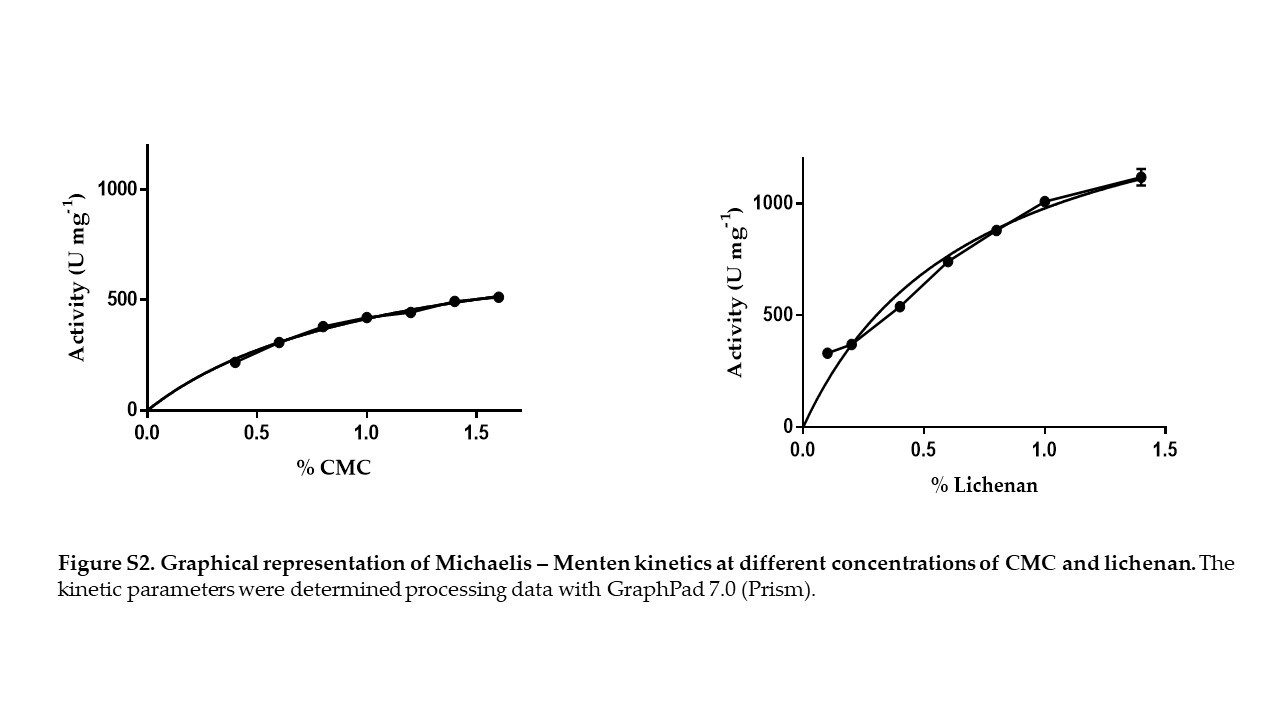

Supplement: Supplementary file 1 [file ijms-24-00243-s001.zip › Supplementary Figure S2.jpg]
